# Convergent IGF2 overexpression in pheochromocytoma/paraganglioma: insights from Beckwith–Wiedemann syndrome

**DOI:** 10.1530/ERC-26-0061

**Published:** 2026-05-18

**Authors:** Hussam Alkaissi, Christopher A Febres-Aldana, Sara Talvacchio, Herui Wang, Catherine M Gordon, Karel Pacak

**Affiliations:** ^1^ National Institute of Diabetes and Digestive and Kidney Diseases, National Institutes of Health, Bethesda, Maryland, USA; ^2^ Laboratory of Pathology, Center for Cancer Research, National Cancer Institute, Bethesda, Maryland, USA; ^3^ Eunice Kennedy Shriver National Institute of Child Health and Human Development, National Institutes of Health, Bethesda, Maryland, USA; ^4^ Neuro-Oncology Branch, National Cancer Institute, National Institutes of Health, Bethesda, Maryland, USA; ^5^ Center of Adrenal Endocrine Tumors, AKESO, Prague 5, Czech Republic; ^6^ Faculty of Medicine, Palacky University, Olomouc, Czech Republic; ^7^ Faculty of Medicine, 5th Department of Medicine, Comenius University, Bratislava, Slovakia

**Keywords:** Beckwith–Wiedemann syndrome, pheochromocytoma, paraganglioma, IGF2, imprinting disorders, methylation

## Abstract

Beckwith–Wiedemann syndrome (BWS) is an imprinting disorder characterized by overgrowth and tumor predisposition, caused by dysregulated expression of genes on chromosome 11p15.5. An association between BWS and pheochromocytoma/paraganglioma (PPGL) has been suggested in isolated case reports over the past fifty years, but the molecular basis for this link remains unclear. We identified four patients with BWS who developed metastatic PPGL and investigated IGF2 pathway activation in these tumors and in PPGL across various genotypes. Pan-cancer transcriptomic analysis of The Cancer Genome Atlas (TCGA) demonstrated that PPGL overexpresses *IGF2*, with pseudohypoxic tumors exhibiting higher expression compared to other molecular clusters. Loss of heterozygosity and loss of imprinting at 11p15.5 partially explain this overexpression, with PPGL additionally demonstrating globally elevated expression of imprinted genes compared to most other tumor types, suggesting a broader relaxation of genomic imprinting. Cognate receptor profiling revealed that PPGLs are equipped to respond to IGF2 signaling, with high expression of IGF1R and insulin receptor isoform A (IR-A). Immunohistochemistry confirmed IGF2 protein overexpression in both BWS-associated and genotypically diverse sporadic PPGLs. Our results indicate that IGF2 overexpression is a convergent molecular feature of PPGL across genotypes and suggest the IGF2 pathway as a potential diagnostic and therapeutic target.

## Introduction

Beckwith–Wiedemann syndrome (BWS) is a rare tumor predisposition and overgrowth syndrome with complex genetics, involving the chromosome 11p15.5 locus. BWS is characterized by macrosomia, macroglossia, hemihypertrophy, and a predisposition to various embryonal tumors, including Wilms tumor, hepatoblastoma, neuroblastoma, and adrenocortical carcinoma (1, 2). The hallmark of BWS-related malignancies is overexpression of the growth factor insulin-like growth factor 2 (*IGF2*) and loss of the cell cycle regulator *CDKN1C* (coding p57 protein). Both *IGF2* and *CDKN1C* genes are located on the 11p15.5 locus and are imprinted (1, 3). The maternal allele of 11p15.5 teleologically aims to control growth of the fetus and placenta by inhibiting IGF2 through long non-coding RNA (lncRNA) H19 hypomethylation and by expressing the cell cycle regulator CDKN1C while suppressing its inhibitor, the lncRNA KCNQ1OT1, through methylation (3, 4, 5). The paternal allele does the opposite, de-repressing IGF2 by methylating its inhibitor H19 and suppressing CDKN1C through expression of its inhibitor KCNQ1OT1. This balance is achieved through two distinct imprinting control regions (ICRs). ICR1 controls H19 methylation in the paternal allele, and ICR2 controls KCNQ1OT1 methylation in the maternal allele (4). The genetic/epigenetic landscape of BWS is complex, encompassing several molecular mechanisms that disrupt the normal balance between maternal and paternal alleles, including loss of methylation at ICR2 (resulting in low CDKN1C), gain of methylation at ICR1 (resulting in high IGF2), paternal uniparental disomy of 11p15.5, and pathogenic variants in *CDKN1C* (6). Such complexity is further compounded by the fact that BWS can arise from germline or somatic mosaic alterations; thus, the spatiotemporal distribution of the molecular variant alters the phenotype and complicates the genetic confirmation (1).

Pheochromocytomas and paragangliomas (PPGLs) are rare, highly heritable neural crest-derived tumors of adrenal medulla and extra-adrenal autonomic paraganglia, respectively (7, 8, 9). Approximately 40% of PPGLs harbor germline pathogenic variants, and 30% harbor somatic variants (10). To date, PPGL susceptibility genes have been categorized into three molecular clusters: pseudohypoxia-driven (e.g. variants in genes controlling the Krebs cycle such as *SDHx*, *FH*, or the hypoxia signaling pathway such as *VHL*, *EPAS1*, and *EGLN1/2*), kinase-signaling (e.g. *RET*, *NF1*, *HRAS*, *TMEM127*, and *MAX*), and Wnt-signaling (e.g. *MAML3* fusions and *CSDE1*) (10). Up to 30% of PPGLs harbor no known driver; thus, despite advances in our understanding of PPGL genetics, the downstream effectors driving tumorigenesis in some tumors remain incompletely characterized. A significant proportion of PPGLs may result in metastasis, with certain genotypes associated with higher risk, such as *SDHB*, *MAML3* fusions, and somatic alterations in *EPAS1* and *ATRX/TERT* (11, 12, 13).

A potential association between BWS and PPGL has been suggested by isolated case reports only. However, whether this association reflects a true mechanistic link or coincidental co-occurrence has not been systematically examined (14, 15, 16, 17, 18, 19, 20, 21). Herein, we report a series of four cases of metastatic PPGL in patients with BWS. We hypothesize that BWS and PPGL may converge on a shared molecular phenotype, namely, the overexpression of insulin-like growth factor 2 (IGF2), albeit through distinct genetic mechanisms. In addition, we provide preliminary immunohistochemical evidence supporting this model across other PPGL molecular subtypes.

## Methods

### Patient identification and clinical characterization

Four patients with clinical features consistent with BWS and PPGL were identified in a cohort of 1,150 patients with PPGL, evaluated over the past 25 years in our center. From the original cohort, 101 patients had genetic testing beyond a targeted panel (40 germline whole genome sequencing; 68 germline and tumor whole exome sequencing) done as a part of clinical care. Clinical features and radiological findings are reported. Histological tissues were available from two patients’ tumors, allowing immunohistochemical analysis.

### Pan-cancer and PPGL-specific gene expression analysis

To examine expression of genes at the 11p15.5 locus across human cancers, we queried GEPIA2 and GEPIA3 (Gene Expression Profiling Interactive Analysis; https://gepia3.bioinfoliu.com) (22, 23). Expression levels of *IGF2*, *H19*, *CDKN1C*, and *KCNQ1OT1* across all available tumor types were visualized as a heatmap of log_2_(TPM + 1) values. To assess *IGF2* expression across PPGL molecular subtypes, we utilized PPGLomics (https://alkaissilab.shinyapps.io/PPGLomics), which integrates transcriptomics data from PPGL consortium datasets with molecular cluster annotations specific to PPGL (10, 12, 24). Tumors were classified into three groups: pseudohypoxia (Cluster 1), kinase signaling (Cluster 2), and Wnt-altered (Cluster 3). *IGF2* expression (log_2_ (TPM + 1)) was compared across clusters and visualized as box plots. Kruskal–Wallis test was used to compare expression across the groups. To investigate the predominant isoform of insulin receptor (INSR) expressed in tumors with high IGF2 expression, we queried the TCGA database through the GEPIA2 platform.

To evaluate receptor expression relevant to IGF2 signaling across human cancers, we queried The Cancer Genome Atlas (TCGA) pan-cancer dataset for INSR, IGF1R, and IGF2R. IGF2 binds INSR with preferential affinity for isoform A (IR-A, INSR-002), as well as IGF1R, both of which mediate its mitogenic signaling. In contrast, IGF2R functions as a scavenger receptor that internalizes and degrades IGF2, thereby attenuating its bioavailability. Median tumor expression (log_2_ TPM) was extracted for 33 TCGA cancer types and ranked from highest to lowest for each receptor.

### Immunohistochemistry

Formalin-fixed paraffin-embedded (FFPE) tissue sections were obtained from PPGL tumors with known driver mutations (germline *SDHB*, *n* = 2; somatic *EPAS1*, *n* = 1; germline *RET*, *n* = 1; somatic *UBTF::MAML3* fusion, *n* = 1) and from two patients in the BWS-PPGL cohort with available tissue (Patient A: intestinal PGL; Patient D: hepatic metastasis). Placental tissue was used as a positive control for *IGF2* expression, and normal adrenal gland as a negative control.

Consecutive 5–7 μm sections were prepared for parallel staining with chromogranin A (to confirm neuroendocrine differentiation and assess colocalization) and IGF2. Heat-induced epitope retrieval (HIER) was performed using citrate buffer (pH 6.0). Endogenous peroxidase activity was quenched using 0.3% H_2_O_2_, and non-specific binding was blocked using Background Punisher (Biocare Medical, #BP974, USA) according to manufacturer’s instructions. Antibodies against chromogranin A (Thermo Fisher Scientific, #PA5-77917, USA) and IGF2 (Thermo Fisher Scientific, #MA5-17096) were diluted in SignalStain Antibody Diluent (Cell Signaling Technology, #8112, USA) at 1:200 dilution. Sections were incubated with primary antibodies overnight at 4°C.

Following washes, sections were incubated with horseradish peroxidase (HRP)-conjugated secondary antibodies: SignalStain Boost IHC Detection Reagent (anti-rabbit, Cell Signaling Technology #8114; anti-mouse, Cell Signaling Technology #8125). Signal was developed using 3,3′-diaminobenzidine (DAB) as chromogen. Slides were counterstained with Mayer’s hematoxylin, dehydrated, and cover-slipped, and whole-slide images were obtained.

### ICR1 methylation and 11p15.5 imprinting analysis

The H19/IGF2 imprinting control region 1 (ICR1) was defined using OMIM-curated GRCh38 coordinates (chr11:1,998,202–2,003,509). DNA methylation data for the TCGA-PCPG cohort were retrieved from the UCSC Xena browser. Probes mapping within the ICR1 locus were identified by genomic position, yielding 39 CpG sites; seven probes (cg06982169, cg25574978, cg19177307, cg19024989, cg01585333, cg22259242, and cg27372170) were excluded due to the absence of methylation data from the TCGA-PCPG HM450 dataset. ICR1 methylation was summarized per sample as the mean beta value across the 39 retained probes.

Gene expression data for H19 and IGF2 of corresponding cases were retrieved from UCSC Xena browser as log_2_(RSEM + 1) normalized counts. A normal adrenal methylation reference was derived from three matched normal tissue samples included in the TCGA-PCPG dataset (mean beta = 0.554 ± 0.020 SD), consistent with the expected monoallelic methylation pattern of a normally imprinted locus. Copy number alteration at chromosome 11p15 locus TCGA-PCPG is downloaded from cBioPotal (25). Multi-track visualization was performed in R using the ComplexHeatmap package.

### Whole exome and RNA sequencing

DNA and RNA were extracted from formalin-fixed paraffin-embedded (FFPE) tumor tissue blocks. Whole exome sequencing (WES) and RNA sequencing (RNA-seq) were performed as previously described (26). Briefly, WES was performed on paired tumor and germline DNA to identify somatic single-nucleotide variants, indels, and copy number alterations. From tumors with WES and CNV data, 5 cases with LOH at 11p15.5 vs maintained heterozygosity were selected for further comparison. For the loss of heterozygosity (LOH) analysis, allele-specific copy number profiles were generated from WES data using the Sequenza algorithm (27), which jointly models B allele frequency and depth ratio from matched tumor–normal pairs to infer allele-specific copy number states, tumor purity, and ploidy. LOH at a given locus was defined by a B allele frequency deviating from the expected heterozygous value of 0.5, consistent with loss of one parental allele. Statistical analyses were performed using GraphPad Prism (version 10.2.2). Comparisons between tumors with 11p15.5 LOH and those with retained heterozygosity were performed using the Mann–Whitney *U* test, given the small sample size (*n* = 5 per group) and non-parametric distribution of expression values.

### Pan-cancer imprinted genes transcriptomic analysis

A list of human imprinted genes was compiled from the GeneImprint database (link: https://www.geneimprint.com), categorized by parental allele of expression into 80 paternally expressed and 43 maternally expressed genes spanning 19 chromosomes. Pan-cancer gene expression data were retrieved from the UCSC Xena platform (log_2_-normalized, cross-cancer batch-corrected; *n* = 11,060 samples, 33 tumor types), accessed using the UCSCXenaTools R package. The expression matrix was filtered to include imprinted genes present in the dataset (available data from 51 paternally expressed genes, and 37 maternally expressed genes). Mean expression per gene per cancer type was computed across all samples, and *Z*-scores were calculated across all 33 cancer types. Paternal and maternal *Z*-scores per cancer type were plotted as a scatter plot.

## Results

### Patient characteristics

#### Patients A and B

**Figure 1:**
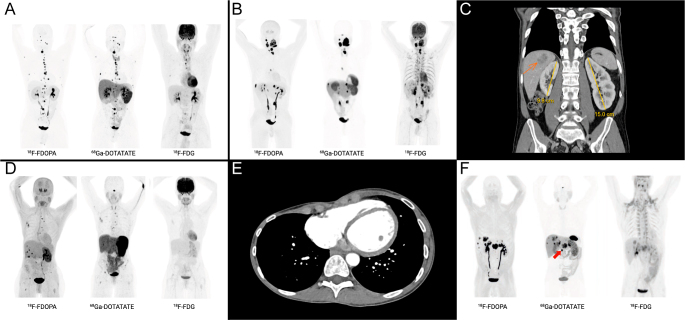
Imaging of metastatic pheochromocytoma/paraganglioma (PPGL) in patients with Beckwith–Wiedemann syndrome (BWS). (A and B) Maximum intensity projection (MIP) images from ^18^F-FDOPA (left), ^68^Ga-DOTATATE (center), and ^18^F-FDG (right) PET scans, demonstrating metastatic disease burden in Patients A and B, respectively. (C) Coronal CT scan from Patient B, demonstrating left renal hemihypertrophy with over 5 cm difference in kidney length (left kidney 15.0 cm vs right kidney 9.8 cm; normal variation <2 cm). Note the metastatic PPGL lesion in the liver (arrow). (D and E) PET scans and CT scan of Patient C, showing metastatic pheochromocytoma. Hemihypertrophy (E) of the chest is seen. (F) PET scans of Patient D. Across all patients, ^18^F-FDOPA PET/CT demonstrated superior lesion detection compared to the other modalities. Notably, in Patient D, a single pancreatic head lesion (red arrow) was detected only on ^68^Ga-DOTATATE PET/CT.

Patients A and B were siblings who were diagnosed with Beckwith–Wiedemann syndrome (BWS) in neonatal period and developed metastatic paraganglioma in adulthood. Both harbor the same germline pathogenic variant in *CDKN1C* (c.688C>T, p.Gln230Ter) with normal ICR1/ICR2 methylation studies. They also carry pathogenic *SDHB* frameshift variants (c.713del, p.Phe238Serfs*10). Patient A is a 29-year-old male with metastatic PGL arising from the left carotid body and organ of Zuckerkandl, with metastases to bones, mesentery, and paraspinal region (Fig. 1A). BWS was diagnosed at birth based on hypoglycemia, omphalocele, macroglossia, left-sided hemihypertrophy, and diaphragmatic hernia; then developed thoracic neuroblastoma at age 1. PGL was diagnosed at age 23. Biochemically, the patient exhibited a normal plasma normetanephrine of 0.81 nmol/L (normal <0.9) and metanephrine <0.5 nmol/L. Surgical resection of a mesenteric lesion confirmed a paraganglioma. He received four cycles of ^177^Lu-DOTATATE with stable disease at 6-year follow-up. Patient B is a 42-year-old male with metastatic head and neck PGL (HNPGL), including bilateral glomus vagale and left carotid body PGL, with metastases to bones, retroperitoneum, lymph nodes, and liver (Fig. 1B). BWS was diagnosed in infancy based on macroglossia, umbilical hernia, and left-sided hemihypertrophy, which was noted on examination and radiological images (Fig. 1C). HNPGL was discovered incidentally during evaluation of obstructive sleep apnea. Biochemically, he exhibited a mixed noradrenergic/dopaminergic phenotype with markedly elevated plasma normetanephrine (7.30 nmol/L; normal <0.90) and dopamine (7.34 nmol/L; normal <0.19). In both patients, ^18^F-FDOPA PET/CT showed higher lesion detection compared to ^68^Ga-DOTATATE PET/CT (Fig. 1A and B).

#### Patient C

Patient C is a 45-year-old female who presented with metastatic pheochromocytoma with metastases to lungs, bones, and liver (Fig. 1D). BWS was clinically diagnosed at age 44 based on the history of bilateral Wilms tumors resected at age 6, macroglossia, and left-sided hemihypertrophy (Fig. 1E). Testing was negative for *CDKN1C* variants with normal IC1/IC2 methylation. At age 34, she was diagnosed with a 10 cm right pheochromocytoma. A 12-gene hereditary PPGL panel was negative. Eleven years after resection of the primary tumor, the patient developed metastatic disease. Biochemically, she exhibited a mixed adrenergic phenotype with elevated plasma metanephrine (0.85 nmol/L; normal <0.5) and normetanephrine (2.2 nmol/L; normal <0.9). Similar to the first two patients, ^18^F-FDOPA PET/CT was more sensitive in lesion detection than ^68^Ga-DOTATATE PET/CT, with minimal ^18^F-FDG uptake (Fig. 1D). She received ^131^I-MIBG resulting in stable disease for 10 years.

#### Patient D

Patient D is a 31-year-old male who presented with metastatic pheochromocytoma discovered during hypertensive crisis (systolic blood pressure >230 mmHg). Imaging revealed an aortocaval mass, bilateral adrenal masses, and hepatic metastases. BWS was diagnosed at birth based on macroglossia and hyperinsulinemic hypoglycemia. He underwent leg lengthening surgery at age 10 for lateralized overgrowth. Medical records suggest hepatic tumor resection in infancy, though pathology confirming hepatoblastoma is unavailable. Germline *CDKN1C* testing was negative. A variant of uncertain significance in *SDHD* (c.278A>G, p.Tyr93Cys) was identified; liver biopsy confirmed metastatic PPGL with retained SDHB protein expression. Biochemically, he exhibited a mixed adrenergic phenotype with normetanephrine of 18.3 nmol/L (normal <0.61), metanephrine of 1.83 nmol/L (normal <0.31), and an elevated dopamine level at 4.87 nmol/L (normal <0.16), yet he remained asymptomatic. PET/CT showed ^18^F-FDOPA detecting more hepatic lesions while ^68^Ga-DOTATATE uniquely identified a pancreatic head lesion (Fig. 1D). After 8 cycles of CVD chemotherapy, he enrolled in an axitinib trial achieving 33% tumor reduction with sustained response at 2.5 years. The clinical features are summarized in Table 1. Eight additional cases of BWS and mostly bilateral, recurrent, and metastatic PPGL had been reported in the literature, as early as in 1976 by Wiedemann’s group (20). Those cases are summarized in Supplementary Table 1 (see section on Supplementary materials given at the end of the article).

**Table 1:** Clinical characteristics of patients with BWS and PPGL.

Patient	A	B	C	D
BWS features	Macroglossia, umbilical hernia, left-sided hemihypertrophy neonatal hypoglycemia, omphalocele, neuroblastoma	Macroglossia, umbilical hernia, left-sided hemihypertrophy	Macroglossia, left-sided hemihypertrophy, Wilms tumor	Macroglossia, neonatal hypoglycemia, hemihypertrophy
Primary PPGL tumor	CBT, OZ	CBT, VGL	PCC	PCC
Age at PPGL diagnosis	23	42	34	31
Sex	Male	Male	Female	Male
Genetics	*SDHB* c. 713delT, p. Phe238Sers*10); IC1 and IC2 methylation normal, *CDKN1C* c.688C>T, p. Gln230*		Negative for 12 PPGL panel; *CDKN1C* negative, IC1 and IC2 methylation normal	*SDHD* VUS c. 278A>G, p. Tyr93Cys; *CDKN1C* negative
Biochemical profile	Metanephrines normal; CgA1.2× elevated	NMN 8× elevated, DA 37× elevated, 3-methoxytyramine 11×	NMN 2.4× elevated, MN 1.7× elevated, 3-methoxytyramine 1.15× elevated, CgA 1.16× elevated	NMN 30× elevated, MN 6× elevated, DA 30× elevated, 3-methoxytyramine 13× elevated, CgA 20× elevated
Treatments for PPGL	Surgery primary, ^177^Lu-DOTATATE	CVD	Surgery of primary tumor, XRT, ^131^I-MIBG	CVD, axitinib

BWS, Beckwith–Wiedemann syndrome; CBT, carotid body tumor; *CDKN1C*, cyclin-dependent kinase inhibitor 1C; CgA, chromogranin A; CVD, cyclophosphamide, vincristine, dacarbazine; DA, dopamine; E, epinephrine; ICR1/ICR2, imprinting control region 1/2; MN, metanephrine; mPCC, metastatic pheochromocytoma; mPGL, metastatic paraganglioma; mPPGL, metastatic pheochromocytoma and paraganglioma; NE, norepinephrine; NMN, normetanephrine; OZ, organ of Zuckerkandl; PCC, pheochromocytoma; PGL, paraganglioma; *SDHB*, succinate dehydrogenase subunit B; *SDHD*, succinate dehydrogenase subunit D; VGL, vagal paraganglioma; VUS, variant of uncertain significance; XRT, external beam radiation therapy.

### The 11p15.5 locus shows selective *IGF2* overexpression in PPGL

**Figure 2:**
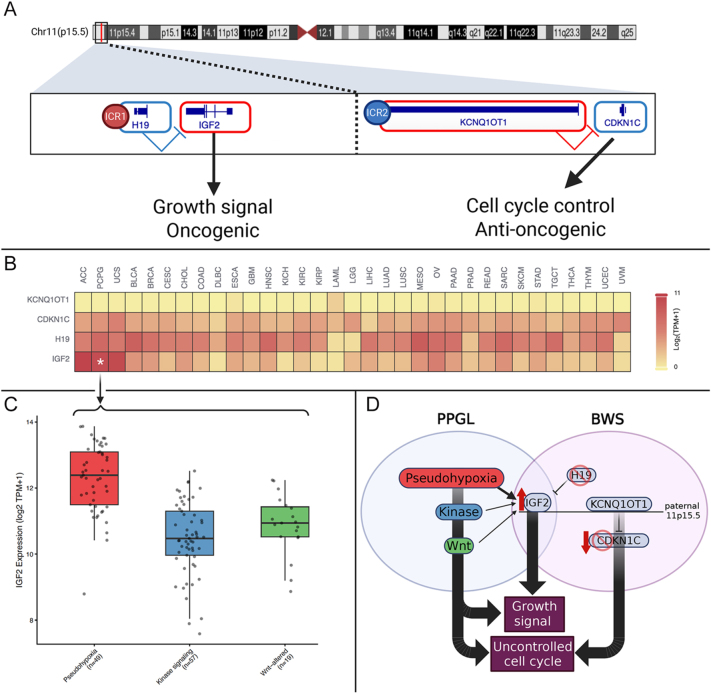
The 11p15.5 imprinted locus and IGF2 expression across cancers and pheochromocytoma/paraganglioma (PPGL) subtypes. (A) Schematic of the 11p15.5 locus showing the two imprinted domains. The ICR1-regulated domain contains *H19* (maternally expressed) and *IGF2* (paternally expressed), with IGF2 functioning as a growth-promoting oncogene. The ICR2-regulated domain contains *KCNQ1OT1* (paternally expressed) and *CDKN1C* (maternally expressed), with CDKN1C (p57) providing cell cycle control and tumor suppression. (B) Heatmap of median of gene expression (log_2_ TPM + 1) for *KCNQ1OT1*, *CDKN1C*, *H19*, and *IGF2* across TCGA tumor types. Asterisk denotes the high IGF2 expression in TCGA-PCPG (PPGL cohort). Other genes of the 11p15.5 locus did not show significant difference among cancers (C) *IGF2* expression across PPGL molecular clusters. Pseudohypoxia-driven tumors (Cluster 1, *n* = 49) show the highest expression, followed by Wnt-altered (Cluster 3, *n* = 19) and kinase signaling (Cluster 2, *n* = 57) tumors. (D) Proposed model of convergent IGF2 overexpression in PPGL and BWS. In PPGL, the three major molecular pathways (pseudohypoxia, kinase signaling, Wnt) lead to IGF2 upregulation ‘pan-PPGL hallmark’. In BWS, germline alterations at 11p15.5 result in IGF2 overexpression (upward red arrow) and/or CDKN1C loss (downward red arrow). Both conditions converge on IGF2-driven growth signaling and loss of cell cycle control, resulting in uncontrolled proliferation.

We examined expression of the major imprinted genes within the 11p15.5 locus (*IGF2/H19*, and *CDKN1C/KCNQ1OT1*) (Fig. 2A) across 33 TCGA tumor types using GEPIA3 analytic platform. Pan-cancer analysis revealed that while *H19*, *CDKN1C*, and *KCNQ1OT1* showed no consistent pattern of differential expression, *IGF2* was markedly and selectively overexpressed in a subset of malignancies (Fig. 2B and Supplementary Fig. 1A). Among all cancers examined, *IGF2* exhibited the highest median expression in adrenocortical carcinoma (ACC; median log_2_(TPM + 1) = 11.2), uterine carcinosarcoma (UCS; 10.59), and pheochromocytoma/paraganglioma (TCGA-PCPG; 10.38). *IGF2* expression in TCGA-PCPG was approximately 21-fold higher than in normal adrenal medulla (*P* = 4.1 × 10^−4^).

To determine whether *IGF2* expression varied by molecular subtype, we analyzed TCGA-PCPG samples using our analytic platform, PPGLomics. *IGF2* expression differed significantly across mRNA expression clusters, with pseudohypoxic tumors exhibiting the highest levels (median = 12.39, *n* = 49) compared to kinase signaling (median = 10.47, *n* = 57) and Wnt-altered (median = 10.94, *n* = 19) subtypes (Kruskal–Wallis *P* = 9.36 × 10^−13^) (Fig. 2C). The selective overexpression of *IGF2*, but not other 11p15.5 transcripts, suggested mechanistic convergence between PPGL and BWS (Fig. 2D), explaining why some individuals with this diagnosis may develop PPGL without carrying a classic genetic driver.

### IGF2 protein is overexpressed in PPGL tissue

**Figure 3:**
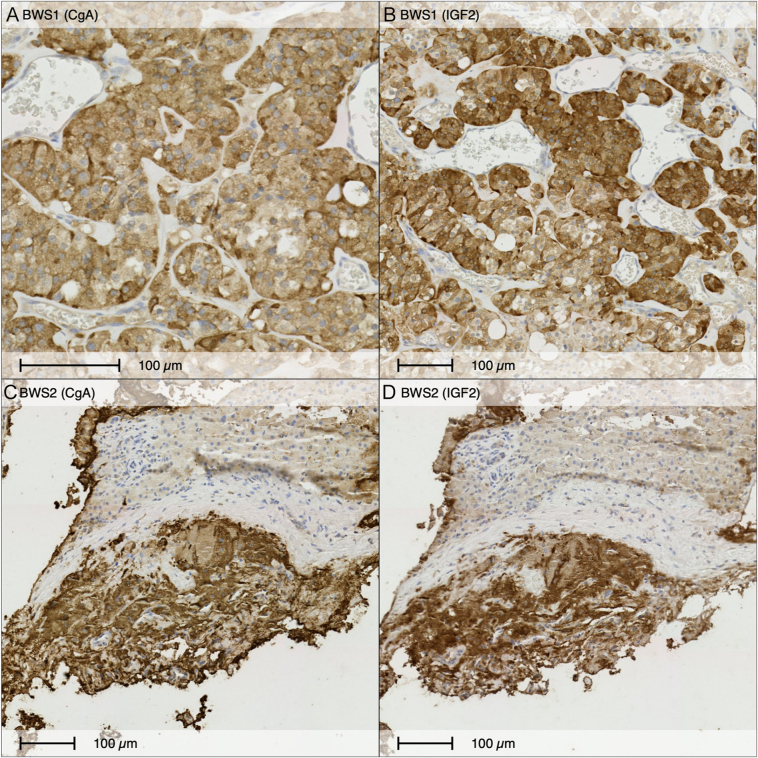
IGF2 immunohistochemistry in pheochromocytoma/paraganglioma (PPGL) in Beckwith–Wiedemann syndrome (BWS) patients. Consecutive sections stained for chromogranin A (CgA, left panels) and IGF2 (right panels) in PPGL tumors form patients with BWS. (A and B) Intestinal paraganglioma form patient A. (C and D) Liver metastatic pheochromocytoma from patient D. Both cases show strong cytoplasmic staining of IGF2 that co-localize to chromogranin A-positive areas. Blood vessels included in the section as an internal negative control. Scale bars: 100 μm.

To validate the transcriptomics findings at the protein level, we performed immunohistochemistry (IHC) on PPGL tissue from two patients with BWS, an intestinal PGL from Patient A and a liver metastatic lesion from Patient D. Consecutive sections were stained for chromogranin A (CgA) as a marker of neuroendocrine tumors and IGF2. IGF2 staining was strongly positive, cytoplasmic, and co-localized with CgA-positive regions (Fig. 3).

**Figure 4:**
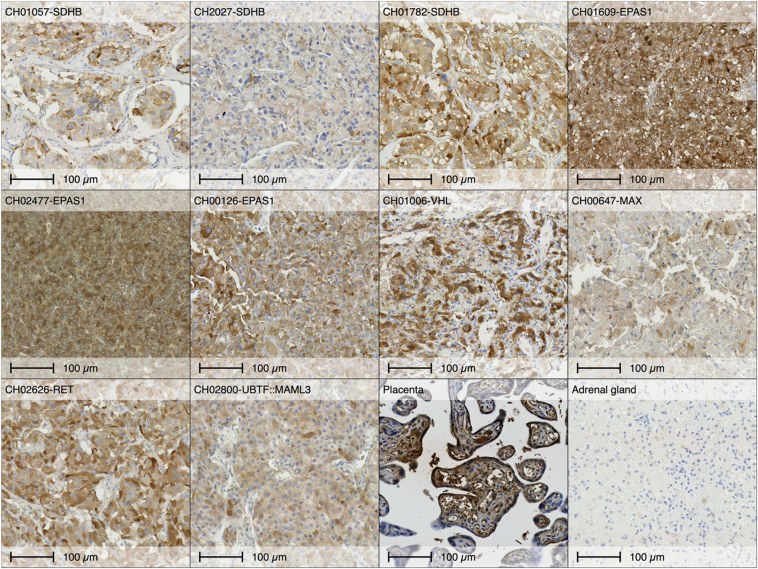
IGF2 immunohistochemistry in pheochromocytoma/paraganglioma (PPGL) across molecular subtypes IGF2 immunostaining in 10 PPGL tumors with known genetic driver. Placenta and normal adrenal gland included as positive and negative control for IGF2, respectively. Scale bars: 100 μm.

To determine whether IGF2 overexpression extends to PPGLs harboring classic driver mutations, we performed IHC on tumors from non-BWS patients with *SDHB*, *EPAS1*, *RET*, and *MAML3* fusion, and apparently sporadic PPGL. Similar to BWS-related PPGL, these tumors demonstrated strong IGF2 staining, whereas normal adrenal medulla and placenta were used as negative and positive controls, respectively (Fig. 4). Patients’ characteristics are summarized in the Supplementary Table 2.

### PPGL tumors favor IGF2 signal transduction over clearance, with high *INSR/IGF1R* and low *IGF2R* expression

All three receptors, *IGF1R*, *IGF2R*, and *INSR* demonstrated expression across TCGA cancers, though with distinct patterns relevant to IGF2 biology in the case of PPGL (Supplementary Fig. 1). The pheochromocytoma and paraganglioma (TCGA-PCPG) cohort exhibited a receptor expression profile favoring IGF2 signaling. PCPG ranked 9th for *IGF1R* expression (median 3.67 log_2_ TPM) (Supplementary Fig. 1B). Conversely, *IGF2R* ranked considerably lower at 20th (median 4.46 log_2_ TPM) (Supplementary Fig. 1C), suggesting diminished capacity for IGF2 clearance relative to other tumor types. PPGL ranked 8th of 33 types of cancers for *INSR* (median 4.14 log_2_ TPM) (Supplementary Fig. 1D). Insulin receptor has two main isoforms, INSR-001 (IR-B), mediating the metabolic effect of insulin, and INSR-002 (IR-A), mediating mitogenic and proliferative signal (28). In addition to binding to insulin, the mitogenic INSR-002 (IR-A) isoform binds to IGF2 in affinity comparable to insulin (28). We analyzed tumors with the highest *IGF2* expression (ACC, UCS, and PCPG) for the different insulin receptor isoforms, and all three show predominance of the mitogenic INSR-002 (IR-A) isoform (Supplementary Fig. 1E). Thus, PPGL appears to be primed to respond to IGF2 proliferative and mitogenic signal (Supplementary Fig. 1F).

### IGF2 overexpression in pseudohypoxic PPGL is associated with 11p15.5 loss of heterozygosity and ICR1 hypermethylation

**Figure 5:**
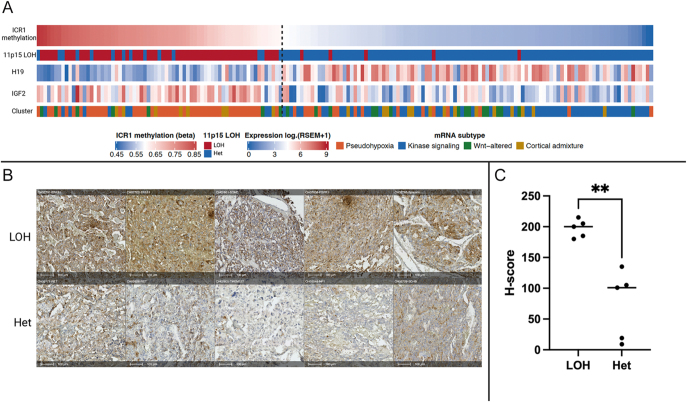
IGF2 overexpression in PPGL is associated with 11p15.5 loss of heterozygosity and ICR1 hypermethylation. (A) Multi-track oncoprint of the TCGA-PCPG cohort (*n* = 173) ordered by descending ICR1 methylation beta value. Tumors with high ICR1 methylation (left of dashed line) show concordant suppression of H19 and overexpression of IGF2 and are enriched in the pseudohypoxic mRNA subtype. 11p15 LOH is the predominant copy number event in this high-methylation group, consistent with preferential loss of the unmethylated maternal allele and retention of the methylated paternal allele. A subset of tumors to the left of the dashed line demonstrates ICR1 hypermethylation without detectable LOH, indicative of loss of imprinting independent of chromosomal loss. (B) Representative IGF2 immunohistochemistry in five PPGLs with 11p15.5 LOH (top row) and five PPGLs with retained heterozygosity (bottom row). Genotypes are indicated on each panel. Scale bars: 100 μm. (C) H-score quantification of IGF2 protein expression demonstrates significantly higher staining in LOH tumors compared to heterozygous tumors (mean H-score 200 vs 101; *P* < 0.001, Mann–Whitney *U* test). Het, retained heterozygosity at 11p15.5; LOH, loss of heterozygosity at 11p15.5; ICR1, imprinting control region 1.

To investigate the mechanism underlying IGF2 overexpression in PPGL, we integrated DNA methylation, copy number alteration, and RNA expression data across 173 primary PPGL from the TCGA-PCPG cohort. Loss of chromosome 11p15.5 is a predominant copy number event in PPGL (Supplementary Fig. 2A), predominantly in pseudohypoxic tumors. Tumors with LOH at 11p15.5 showed elevated ICR1 methylation, consistent with preferential loss of the maternal allele and retention of the methylated paternal copy. This was further supported by suppression of H19 expression and reciprocal overexpression of IGF2 (Fig. 5A). A subset of tumors exhibited loss of imprinting without detectable LOH, suggesting that epigenetic dysregulation of ICR1 can occur independently of chromosomal loss. Patients’ clinical characteristics are summarized in Supplementary Table 3. To validate these findings, we selected 10 PPGL cases (5 with LOH at 11p15.5 and 5 with retained heterozygosity (Supplementary Fig. 2B), for bulk RNA sequencing and immunostaining. IGF2 expression was significantly higher in tumors with LOH at 11p15.5, than in tumors with retained heterozygosity (mean TPM 5,716 vs 1,186; *P* = 0.016) (Supplementary Fig. 2C and D). Immunohistochemistry confirmed higher IGF2 protein expression in LOH cases (Fig. 5B), with significantly elevated H-scores (mean 200 vs 101; *P* < 0.001, Mann–Whitney) (Fig. 5C). Notably, higher IGF2 expression was associated with greater tumor burden as measured by sum of diameters (SOD) (Supplementary Fig. 2E).

### Pan-genomic imprinting relaxation distinguishes PPGL from other cancers

Several imprinted genes are notably expressed in PPGL (e.g. GNAS, DLK1, and RTL1) and are distributed across the genome, prompting us to investigate the expression of all imprinted genes in PPGL using pan-cancer data. Surprisingly, PPGL ranked first among all 33 TCGA cancer types in paternally expressed imprinted gene expression (*Z* = +2.22) and fourth in maternally expressed gene expression (*Z* = +1.16) (Supplementary Fig. 2F). This bimodal elevation, affecting both paternally and maternally expressed genes distributed across the genome, cannot be explained by focal chromosomal loss at 11p15.5 alone, and instead suggests global relaxation of genomic imprinting, warranting further investigation.

## Discussion

We present a case series of metastatic PPGL arising in patients with BWS, leading to the hypothesis that these two conditions converge on a shared molecular signature: overexpression of IGF2. The 11p15.5 locus harbors two imprinted gene clusters with opposing functions, the ICR1-regulated *IGF2/H19* domain, where IGF2 acts as a growth-promoting oncogene, and the ICR2-regulated *KCNQ1OT1/CDKN1C* domain, where *CDKN1C* encodes the tumor suppressor p57, regulating cell cycle. In BWS, disruption of imprinting at this locus drives *IGF2* overexpression and/or *CDKN1C* loss, producing the characteristic overgrowth phenotype and tumor predisposition. In pan-cancer transcriptomic analysis, PPGL ranked among the three tumor types with the highest IGF2 expression, alongside adrenocortical carcinoma and uterine carcinosarcoma. Within PPGL, expression was highest in pseudohypoxic (Cluster 1) tumors, with lower but detectable expression across kinase-signaling and Wnt-altered clusters. IHC confirmed IGF2 protein overexpression across diverse PPGL genotypes, as well as in BWS-associated cases, with higher expression in tumors with LOH at 11p15.5 locus.

Despite its higher circulating levels in physiological state, IGF2 biological role is still elusive when compared to IGF1 (29). IGF2 functions as a potent mitogen, signaling through the IGF1 receptor (IGF1R) and insulin receptor (INSR), specifically isoform A (IR-A) to activate PI3K/Akt and MAPK pathways (29). Clinically, extreme IGF2 overexpression can manifest as tumor-induced hypoglycemia (Doege-Potter syndrome), typically seen with large mesenchymal tumors secreting incompletely processed ‘big’ IGF2 (30). Notably, we did not observe hypoglycemia in our BWS-PPGL cohort, nor has this been a reported feature of PPGL. This may indicate that IGF2 acts predominantly through local autocrine/paracrine mechanisms in PPGL rather than achieving systemic concentrations sufficient for metabolic effects, as shown previously by Gelato and Vassalotti (31). Alternatively, the hyperglycemic milieu induced by catecholamine excess may mask or counteract any hypoglycemic effect of circulating IGF2.

The role of *IGF2* overexpression in BWS-associated embryonal tumors is well established. Loss of imprinting at the 11p15.5 locus, leading to biallelic *IGF2* expression, was first demonstrated in Wilms tumor, and subsequently confirmed in hepatoblastoma and other BWS-associated malignancies (32, 33, 34). *IGF2* overexpression is recognized as the central molecular driver of several pediatric and non-pediatric tumors, including those in BWS (29, 35). Our findings suggest that PPGL may be added to this list of IGF2-driven malignancies.

The connection between IGF2 and tumors of neural crest origin is not new. Neuroblastoma, which shares embryonic origins with PPGL through the sympathoadrenal lineage, has long been known to express and rely on IGF2 signaling (36, 37, 38). Similar to our observation of higher IGF2 in pseudohypoxia-related PPGL, *IGF2* is associated with *EPAS1* (encoding hypoxia inducible factor-2α, or HIF-2α) in neuroblastoma. Both IGF2 and HIF-2α are early markers of sympathetic neurons, and expression of HIF-2α is partly controlled by IGF2 in this context (38). This shared developmental origin prompted earlier investigators to examine IGF2 expression in PPGL (31, 36, 39, 40). While those studies provided earlier clues to the role of IGF2 in PPGL, these studies predated the genomic era and modern molecular classification of PPGL, thus limiting its interpretation. Our analysis, stratified by molecular cluster, demonstrates that IGF2 overexpression is a pan-PPGL phenomenon, with higher levels in pseudohypoxic tumors.

Possible mechanisms for IGF2 overexpression in PPGL include somatic alterations at 11p15.5 (including loss of imprinting or loss of heterozygosity affecting the maternal allele), or indirect transcriptional activation. We show in the PPGL cohort that tumors with higher IGF2 have loss of heterozygosity at 11p15.5 locus, a finding validated by the TCGA-PCPG cohort. Furthermore, TCGA cohort tumors with higher *IGF2* expression have higher methylation at ICR1 with suppression of *H19* expression, indicating the possibility of selection for clones with maternal loss of 11p15.5. While loss of imprinting and heterozygosity is an attractive explanation, lessons from Wilms tumor suggest that IGF2 overexpression can occur through mechanisms entirely independent of imprinting status. Wilms tumors exhibit 30–57% LOH at the 11p15.5 locus with a significant proportion showing loss of imprinting, yet IGF2 mRNA levels are universally elevated (greater than 5-fold compared to adjacent normal kidney tissue) even in tumors that maintain normal imprinting and retain both parental alleles (41). This universality of IGF2 overexpression in Wilms tumors indicates alternative mechanisms of IGF2 transcriptional activation that remain to be fully characterized and may apply to PPGL and other tumors. Additional mechanisms that converge on IGF2 upregulation have been identified, including mutations in microRNA processing genes (*DROSHA*, *DGCR8*, *DICER1*) leading to *PLAG1* de-repression, *DIS3L2* loss-of-function, and *LIN28* overexpression, all of which activate IGF2 transcription through distinct upstream pathways independent of 11p15.5 epigenetic alterations (35).

As a pan-PPGL phenomenon, the therapeutic implications of targeting IGF2 in PPGL merit consideration. Targeting the IGF signaling axis has been attempted in other IGF2-driven malignancies, such as adrenocortical carcinoma, where the IGF1R inhibitor linsitinib was trialed but did not demonstrate clinical benefit in a phase III trial (42). The unsatisfactory results regarding linsitinib in ACC may reflect parallel signaling through IR-A, which can also transduce IGF2 signals and was unopposed. Alternative strategies include dual IGF1R/IR-A inhibitors, or IGF2-neutralizing antibodies (e.g. xentuzumab), or approaches targeting IGF2 expression directly (43).

It has not escaped our notice that IGF2 overexpression in PPGL may reflect a broader phenomenon of global imprinting relaxation. The coordinate de-repression of multiple imprinted loci represents an understudied dimension of PPGL tumor biology that warrants further investigation (44). Such a mechanism could provide an insight into the ‘parent-of-origin’ effect observed in *SDHD*, *SDHAF2*, and *MAX*-associated hereditary PGL syndromes, where pathogenic variants exhibit penetrance primarily when inherited paternally. The implications of a generalized paternal genomic bias in PPGL tumorigenesis, including its potential impact on other imprinted loci and therapeutic vulnerabilities, are to be further explored.

Our cohort of BWS-PPGL patients carried direct clinical implications for the management of patients with BWS. Clinicians should maintain a low threshold for consideration of PPGL in BWS patients presenting with adrenal or retroperitoneal masses, or with symptoms suggestive of catecholamine excess. Furthermore, given the pattern of aggressive and metastatic disease observed in our cohort and reported cases, BWS-associated PPGL should be regarded as a high-risk phenotype warranting lifelong postoperative surveillance.

Several limitations warrant acknowledgment. First, the rarity of BWS-PPGL cases raises the question of whether this represents a coincidental finding; however, we address this concern by demonstrating that PPGL broadly overexpresses IGF2, one of the principal oncogenic drivers in BWS-related tumors, making PPGL a biologically plausible tumor type within the BWS spectrum. Second, tumor tissue was available from only two patients as histology slides, which precluded somatic mutation and ICR epimutation analysis. Third, Patient C carried a clinical BWS diagnosis without a confirmatory molecular alteration, reflecting the well-recognized diagnostic complexity of BWS, where mosaicism and epimutations can produce a full phenotype undetectable by standard assays. Fourth, co-occurring genetic variants complicate genotype-phenotype causality. Patients A and B carry both *CDKN1C* and *SDHB* pathogenic variants, and the contribution of each cannot be fully disentangled; we propose that *CDKN1C* loss acts as a cooperating modifier consistent with the multi-hit model of tumorigenesis of other organs, as exemplified by *APC* and *KRAS* cooperation in colorectal cancer and *BRAF* and *PTEN* loss in melanoma (45, 46, 47). For Patient D, the *SDHD* variant (p.Tyr93Cys) is present in 0.12% of individuals of African ancestry in gnomAD, consistent with a population polymorphism rather than a pathogenic PPGL driver. Finally, our findings remain descriptive; the functional consequences of IGF2 overexpression in PPGL and the effect of its inhibition require validation in cell line or animal models before biological and therapeutic conclusions can be drawn.

## Conclusion

We provide evidence that BWS and PPGL converge on IGF2 overexpression. Rather than viewing BWS-associated PPGL as a rare curiosity, we propose that BWS provides a window into fundamental PPGL biology, highlighting IGF2 as a shared molecular feature across this heterogeneous tumor type. Pan-cancer analysis shows that PPGL is primed to respond to IGF mitogenic signal, by expressing IGF1R and IR-A. The overexpression of IGF2 in PPGL across the different molecular clusters suggests potential for genotype-agnostic therapeutic targeting and a pathologic marker. Further investigation into the mechanisms driving IGF2 expression in PPGL and the functional consequences of its inhibition is warranted.

## Supplementary materials



## Declaration of interest

The authors declare that there is no conflict of interest that could be perceived as prejudicing the impartiality of the work reported.

## Funding

This research was in part supported by the Intramural Research Program of the National Institute of Diabetes and Digestive and Kidney Diseases (NIDDK), National Cancer Institute (NCI), and *Eunice Kennedy Shriver* National Institute of Child Health and Human Development (NICHD) within the National Institute of Health (NIH). The contributions of the NIH author(s) are considered Works of the United States Government. The findings and conclusions presented in this paper are those of the author(s) and do not necessarily reflect the views of the NIH or the US Department of Health and Human Services.

## Author contribution statement

HA, CMG, and KP designed the study, collected and interpreted data, and drafted the manuscript. HA, CFA, ST, HW, CMG, and KP collected and interpreted data. All authors provided critical review and feedback for the final manuscript.

## Ethics statement

This study was conducted in accordance with the Declaration of Helsinki. The protocol (00-CH-0093) was approved by the NIH Institutional Review Board. Written informed consent was obtained from all patients.
