# Supplementary material for: Convergent IGF2 overexpression in pheochromocytoma/paraganglioma: insights from Beckwith–Wiedemann syndrome
Source: Endocr Relat Cancer. 2026 May 18;33(5):e260061. doi: 10.1530/ERC-26-0061 (PMC13192690; doi:10.1530/ERC-26-0061)
Supplement: Supplementary file 1 [file supplementary_materials.pdf]

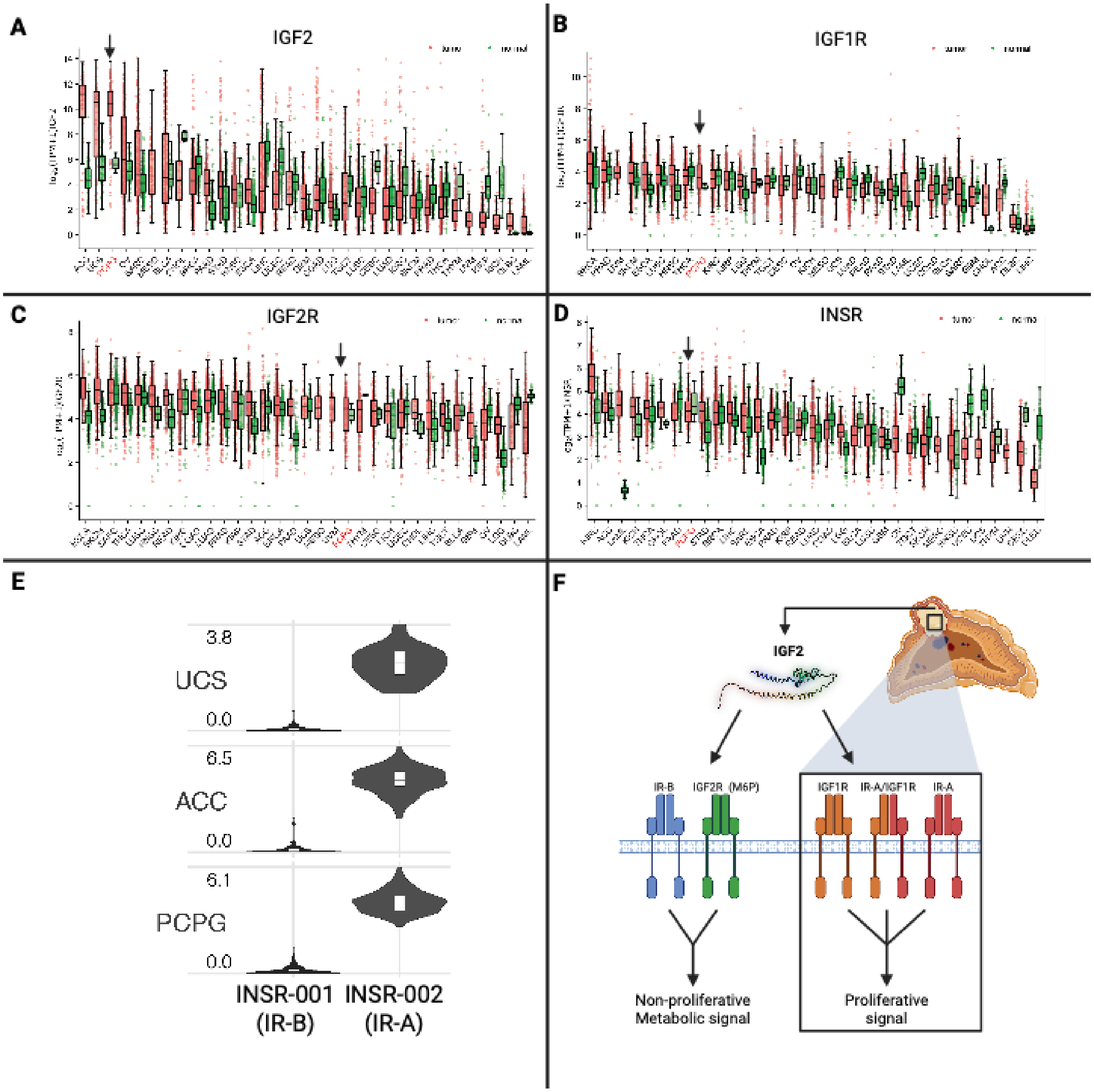

Supplementary Figure 1: IGF2 and its cognate receptors in pheochromocytoma and paraganglioma (PPGL).

Pan-cancer analysis of *IGF2* and its three receptors' expression across TCGA tumor types. **(A)** IGF2 expression showing pheochromocytoma and paraganglioma (TCGA-PCPG) as the third-highest expressing tumor type after adrenocortical carcinoma (ACC) and uterine carcinosarcoma

(UCS). **(B)** *IGF1R* expression, with TCGA-PCPG showing high levels of expression. **(C)** *IGF2R* expression, notably lower in PCPG relative to the proliferative receptors. **(D)** *INSR* expression across cancers. **(E)** Violin plots displaying transcript-level expression of *INSR* isoforms in the three TCGA tumors with highest IGF2 expression (UCS, ACC, PCPG). Across all three malignancies, INSR-002 (encoding IR-A) isoform is the dominantly expressed isoform rather than the metabolic INSR-001 (IR-B). IR-A binds IGF2 with affinity comparable to insulin, whereas IR-B has lower affinity for IGF2. **(F)** Proposed model of autocrine/paracrine IGF2 signaling in pheochromocytoma. Tumor cells produce IGF2 locally, which signals through proliferative receptors (IGF1R, IR-A, and IR-A/IGF1R hybrid receptors) rather than through non-proliferative pathways (IR-B, IGF2R).

Abbreviations: ACC, adrenocortical carcinoma; IGF1R, insulin-like growth factor 1 receptor; IGF2, insulin-like growth factor 2; IGF2R, insulin-like growth factor 2 receptor (mannose-6-phosphate receptor); INSR, insulin receptor; IR-A, insulin receptor isoform A; IR-B, insulin receptor isoform B; M6P, mannose-6-phosphate; TCGA-PCPG, The Cancer Genome Atlas pheochromocytoma and paraganglioma cohort; TPM, transcripts per million; UCS, uterine carcinosarcoma.

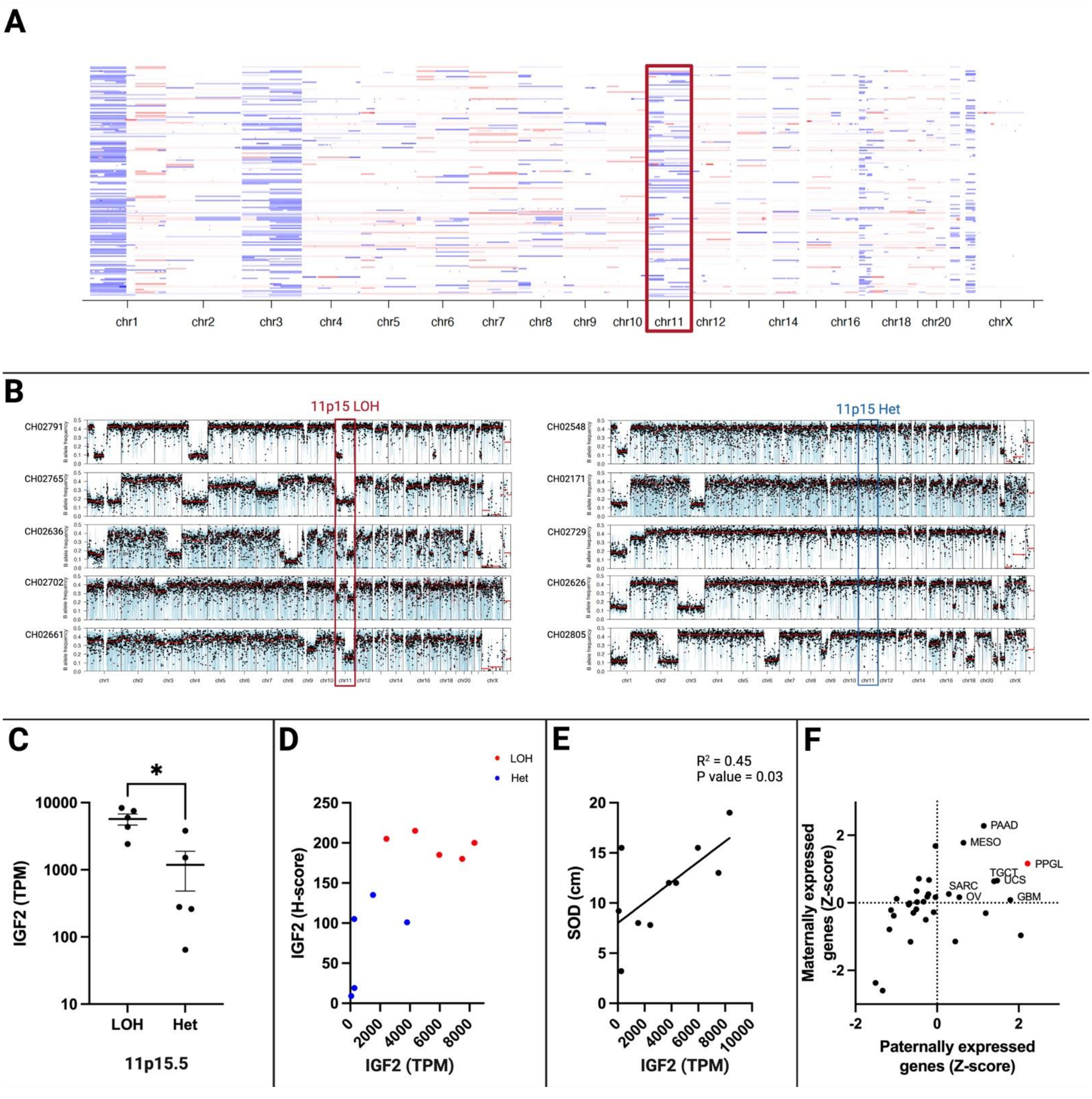

Supplementary Figure 2. (A) Genome-wide copy number alteration heatmap across the TCGA-PCPG cohort (n=173). Recurrent chromosomal losses (blue) are observed at chromosomes 1p, 3, 11, 17, 21, and 22. Chromosome 11 loss is highlighted (red box) and represents one of the most frequent somatic copy number events in this cohort. **(B)** B-allele frequency plots generated using

the Sequenza algorithm for ten representative cases. Five tumors with 11p15.5 LOH (left, red box) and five with retained heterozygosity (right, blue box). **(C)** IGF2 mRNA expression (TPM) from RNA-seq of the same ten cases. LOH tumors demonstrate significantly higher IGF2 expression compared to heterozygous tumors (mean ± SEM: 5,716 vs. 1,186 TPM; p=0.016, Mann-Whitney U test). **(D)** Concordance between IGF2 mRNA expression (TPM) and protein expression (H-score by IHC) across LOH (red) and Het (blue) tumors. **(E)** Simple linear regression demonstrating a positive correlation between IGF2 mRNA expression (TPM) and tumor size, shown as the sum of longest tumor diameters (SOD) (R^2=0.45, p=0.03). **(F)** Pan-cancer z-score analysis of maternally versus paternally expressed imprinted genes across 33 TCGA tumor types. PPGL (red dot) clusters in the upper right quadrant, indicating coordinate overexpression of both maternally and paternally expressed imprinted genes, consistent with global imprinting relaxation. Abbreviations: Het, retained heterozygosity at 11p15.5; LOH, loss of heterozygosity at 11p15.5; PPGL, pheochromocytoma/paraganglioma; SOD, sum of longest diameters; TPM, transcripts per million; GBM, glioblastoma multiforme; MESO, mesothelioma; OV, ovarian serous cystadenocarcinoma; PAAD, pancreatic adenocarcinoma; SARC, sarcoma; TGCT, testicular germ cell tumor; UCS, uterine carcinosarcoma.

Supplementary Table 1: Clinical characteristics of patients with BWS and PPGL (Literature review)

Publication	PPGL Diagnosis Age	Sex	Tumor Site	Metastasis or recurrence	Treatment	Biochemistry	BWS clinical manifestations	BWS molecular testing	PPGL genetic testing
Kalish et al., 2013(1)	18 months	F	Bilateral PCC	No recurrence or metastasis (30 months post-op)	Surgery	Normal plasma NMN/MN	Include as paper says IH dx, not BWS?<br>4 months isolated hemihypertrophy; umbilical hernia	Germline homozygosity for 11p, no methylation defects of ICR1 or ICR2. Somatic: 5% of cells in skin biopsy from larger leg showed homozygosity for	Somatic: mosaic deletions of 8p12, 21q21.1, 22q11.23; mosaic 11p15.3 homozygosity; Germline: negative for

								11p15 (mosaic paternal UPD)	*VHL, SDHB, SDHD, RET*
Caza et al., 2017(2)	15 years	F	Left PCC, para-aortic PGL	Right pheochromocytoma, left retroperitoneal paraganglioma with positive metastatic lymph node	Surgery	Plasma NMN elevate at 2.42 nmol/L (ULN<0.9)	Hemihypertrophy with leg length discrepancy (s/p osteotomy age 15), neonatal hyperglycemia/hyperinsulinemia, cystic pancreatic tumor resected as infant, bilateral optic nerve edema	Germline: No major copy gains/losses. Normal karyotype.	Negative germline on 12-gene PPGL panel
Bemurat et al., 2002 (3)	20 years	F	Bilateral PCC	Follow up not provided	Surgery	24-hour urine total metanephrines 6-7 folds elevation; NE 24 folds elevated. Normal plasma catecholamines.	Hemihypertrophy, Hypoglycemia, omphalocele, hepatomegaly, bilateral mammary adenofibroma	Not provided	Not provided
Baldisserotto et al., 2005(4)	6 years	M	Bilateral PCC	Follow up not provided	Surgery	Elevated urine catecholamines	Hemihypertrophy, nephromegaly, umbilical hernia, hypoglycemia	Not provided	Not provided
Kundal et al., 2023(5)	14 years	F	Bilateral PCC	Follow up not provided	Surgery	Not provided	Pancreatic tumor, lower extremity hemihypertrophy and limb length discrepancy requiring osteotomy, hypoglycemia	Not provided	Not provided
Wilson et al., 2008(6)	8 years	F	Bilateral PCC	No recurrence or metastasis	Surgery (complicated by hypertensive emergency)	Elevated urine catecholamines	Macroglossia, umbilical hernia, hepatosplenomegaly, LVH, hypoglycemia/hyperinsulinemia, periportal liver fibrosis age 3, granulocyte hyperplasia, joint	Loss of methylation at both ICR1 and ICR2; Very high somatic paternal UPD in chromosome 11. Normal karyotype	Germline testing was negative for *SDHD* or *SDHB* variants

							arthritis age 9		
Schnackenburg et al. 1976 (7)	12 years	M	Right PCC	Recurrence after resection, and metastasis to lungs, liver, lymph nodes, femur and brain (died of brain hemorrhage)	Surgery (giant tumor weighing 1.1 kg)	Non-secretory	Macrosomia (birth weight 4600 gram), hemihypertrophy (right side).	Not provided	Not provided
Van den Akker et al. 2002(8)	12 years and 17 years	F	Bilateral PCC	No recurrence or metastasis	Surgery	Noradrenergic (plasma NE 6037 pg/mL, ULN <600.	Hemihypertrophy, macroglossia, breast fibroadenoma, adrenocortical nodular hyperplasia, early puberty.	Methylation patterns for *H19* and *KCNQ1OT1* were normal. Normal karyotype.	Negative germline testing for *RET, VHL* and *NF1* genes.

Abbreviations: BWS, Beckwith-Wiedemann syndrome; H19, H19 imprinted maternally expressed transcript; ICR1/ICR2, imprinting control region 1/2; IH, isolated hemihypertrophy; *KCNQ1OT1*, *KCNQ1* opposite strand/antisense transcript 1; LVH, left ventricular hypertrophy; MN, metanephrine; NE, norepinephrine; NF1, neurofibromatosis type 1; NMN, normetanephrine

Supplementary Table 2. Clinical and pathological characteristics of PPGL cases stained for IGF2 immunohistochemistry (Figure 4), stratified by genetic driver.

Patient	Sex	Age at diagnosis	Location	Tumor behavior	Genotype	DNA Change	Protein change
CH00126	F	26	A-PGL	Metastatic to LN	EPAS1	c.1589C>T	p.Ala530Val
CH02477	F	45	UB-PGL	Primary non-metastatic	EPAS1	c.1591C>T	p.Pro531Ser
CH01609	M	12	A-PGL	Metastatic to bones	EPAS1	c.1603_1624del	p.Met535_Leu542delinsVal
CH00647	M	33	PCC	Multifocal disease	MAX	c.223C>T	p.Arg33Ter
CH02626	F	25	PCC	Primary non-metastatic	RET	c.2753T>C	p.Met918Thr
CH01057	M	15	A-PGL	Primary non-metastatic	SDHB	c.268C>T	p.Arg90Ter
CH2027	F	16	A-PGL	Primary non-metastatic	SDHB	c.380T>G	p. Ile127Ser
CH01782	M	59	A-PGL	Metastatic to liver	SDHB	c.136C>T	p.Arg46Ter

CH02800	F	70	PCC	Metastatic to lungs	UBTF::MAML3	Fusion	
CH01006	M	33	PCC	Primary non-metastatic	VHL	c.713G>A	p.Arg167Gln

Abbreviations: A-PGL, abdominal paraganglioma; F, female; M, male; PCC, pheochromocytoma; PPGL, pheochromocytoma/paraganglioma.

Supplementary Table 3. Clinical, genetic, and molecular characteristics of PPGL cases selected for IGF2 immunohistochemistry validation by 11p15.5 heterozygosity status (Figure 5B).

ID	Sex	Age	Primary tumor	SOD (cm)	Genotype	DNA change	Protein change	11p15.5 LOH	IGF2 (TPM)	IGF2 (H-score)
CH02791	F	68	PCC	19	EPAS1	c.1591C>G	p.Pro531Ala	Yes	8326.8	200
CH02765	M	57	PCC	7.8	Sporadic	NA	NA	Yes	2423.3	205
CH02636	M	73	PCC	15.5	FGFR1	c.1638C>G	p.Asn546Lys	Yes	5971.8	185
CH02702	F	63	A-PGL	12	EPAS1	c.1591C>G	p.Pro531Ala	Yes	4349.7	215
CH02661	M	21	A-PGL	13	SDHD	c.53dup	p.Leu19Serfs*50	Yes	7511.9	180
CH02548	M	71	PCC	15.5	NF1	c.3264delA	p.Glu1089fs*7	No	279.3	19
CH02171	F	34	PCC	8	RET	c.1900T>C	p.Cys634Arg	No	1519.4	135
CH02729	M	14	A-PGL	12	SDHB	c.638T>G	p.Met213Arg	No	3806.8	101
CH02626	F	26	PCC	3.2	RET	c.2752A>G	p.Met918Thr	No	261.1	105
CH02805	F	34	PCC	9.2	TMEM127	c.117_120del	p.Ile41Argfs*39	No	64.3	9

Abbreviations: A-PGL, abdominal paraganglioma; F, female; LOH, loss of heterozygosity at 11p15.5; M, male; NA, not applicable; PCC, pheochromocytoma; SOD, sum of longest diameters; TPM, transcripts per million.

References:

1. Kalish JM, Conlin LK, Mostoufi-Moab S, Wilkens AB, Mulchandani S, Zelley K, et al. Bilateral pheochromocytomas, hemihyperplasia, and subtle somatic mosaicism: the importance of detecting low-level uniparental disomy. Am J Med Genet A. 2013;161A(5):993-1001.

2. Caza T, Manwaring J, Riddell J. Recurrent, bilateral, and metastatic pheochromocytoma in a young patient with Beckwith-Wiedemann syndrome: A genetic link? Can Urol Assoc J. 2017;11(5):E240-E3.

3. Bemurat L, Gosse P, Ballanger P, Tauzin-Fin P, Barat P, Lacombe D, et al. Successful laparoscopic operation of bilateral pheochromocytoma in a patient with Beckwith-Wiedemann syndrome. J Hum Hypertens. 2002;16(4):281-4.

4. Baldisserotto M, Peletti AB, Angelo de Araujo M, Pertence AP, Dora MD, Maciel EO, Gaiger AM. Beckwith-Wiedemann syndrome and bilateral adrenal pheochromocytoma: sonography and MRI findings. Pediatr Radiol. 2005;35(11):1132-4.

5. Kundal D, Leontieva L, Megna JL. Psychiatric Disorder in a Patient With Beckwith-Wiedemann Syndrome: A Case Report. Cureus. 2023;15(6):e40377.

6. Wilson M, Peters G, Bennetts B, McGillivray G, Wu ZH, Poon C, Algar E. The clinical phenotype of mosaicism for genome-wide paternal uniparental disomy: two new reports. Am J Med Genet A. 2008;146A(2):137-48.

7. Schnakenburg KV, Muller M, Dorner K, Harms D, Schwarze EW. Congenital hemihypertrophy and malignant giant pheochromocytoma - a previously undescribed coincidence. Eur J Pediatr. 1976;122(4):263-73.

8. van den Akker EL, de Krijger RR, de Herder WW, Drop SL. Congenital hemihypertrophy and pheochromocytoma, not a coincidental combination? Eur J Pediatr. 2002;161(3):157-60.
